# Rapid viral metagenomics using SMART-9N amplification and nanopore sequencing

**DOI:** 10.12688/wellcomeopenres.17170.2

**Published:** 2023-04-24

**Authors:** Ingra M. Claro, Mariana S. Ramundo, Thais M. Coletti, Camila A. M. da Silva, Ian N. Valenca, Darlan S. Candido, Flavia C. S. Sales, Erika R. Manuli, Jaqueline G. de Jesus, Anderson de Paula, Alvina Clara Felix, Pamela dos Santos Andrade, Mariana C. Pinho, William M. Souza, Mariene R. Amorim, José Luiz Proenca-Modena, Esper G. Kallas, José Eduardo Levi, Nuno Rodrigues Faria, Ester C. Sabino, Nicholas J. Loman, Joshua Quick

**Affiliations:** 1 Faculdade de Medicina da Universidade de São Paulo, Sao Paulo, 05403-000, Brazil; 2 MRC Centre for Global Infectious Disease Analysis, J-IDEA, Imperial College London, London, SW7 2AZ, UK; 3 Instituto de Medicina Tropical, Faculdade de Medicina da Universidade de São Paulo, Sao Paulo, 05403-000, Brazil; 4 School of Biosciences, University of Birmingham, Birmingham, B15 2TT, UK; 5 Department of Zoology, University of Oxford, Oxford, OX1 3SZ, UK; 6 Faculdade de Saúde Pública da Universidade de São Paulo, Sao Paulo, 01246-904, Brazil; 7 World Reference Center for Emerging Viruses and Arboviruses and Department of Microbiology and Immunology, University of Texas Medical Branch, Galveston, TX, 77555, USA; 8 Laboratory of Emerging Viruses, Department of Genetics, Microbiology, and Immunology, Institute of Biology, University of Campinas, Campinas, 13083-862, Brazil; 9 Experimental Medicine Research Cluster, University of Campinas, Campinas, 13083-862, Brazil; 10 DASA, Sao Paulo, 06455-010, Brazil

**Keywords:** RNA virus, metagenomic, nanopore sequencing, genomic surveillance, diagnostic, ZIKV, YFV, SARS-CoV-2

## Abstract

Emerging and re-emerging viruses are a global health concern. Genome sequencing as an approach for monitoring circulating viruses is currently hampered by complex and expensive methods. Untargeted, metagenomic nanopore sequencing can provide genomic information to identify pathogens, prepare for or even prevent outbreaks.

SMART (Switching Mechanism at the 5′ end of RNA Template) is a popular approach for RNA-Seq but most current methods rely on oligo-dT priming to target polyadenylated mRNA molecules. We have developed two random primed SMART-Seq approaches, a sequencing agnostic approach ‘SMART-9N’ and a version compatible rapid adapters  available from Oxford Nanopore Technologies ‘Rapid SMART-9N’. The methods were developed using viral isolates, clinical samples, and compared to a gold-standard amplicon-based method. From a Zika virus isolate the SMART-9N approach recovered 10kb of the 10.8kb RNA genome in a single nanopore read. We also obtained full genome coverage at a high depth coverage using the Rapid SMART-9N, which takes only 10 minutes and costs up to 45% less than other methods. We found the limits of detection of these methods to be 6 focus forming units (FFU)/mL with 99.02% and 87.58% genome coverage for SMART-9N and Rapid SMART-9N respectively. Yellow fever virus plasma samples and SARS-CoV-2 nasopharyngeal samples previously confirmed by RT-qPCR with a broad range of Ct-values were selected for validation. Both methods produced greater genome coverage when compared to the multiplex PCR approach and we obtained the longest single read of this study (18.5 kb) with a SARS-CoV-2 clinical sample, 60% of the virus genome using the Rapid SMART-9N method.

This work demonstrates that SMART-9N and Rapid SMART-9N are sensitive, low input, and long-read compatible alternatives for RNA virus detection and genome sequencing and Rapid SMART-9N improves the cost, time, and complexity of laboratory work.

## Introduction

RNA viruses are responsible for causing a broad range of human and veterinary diseases. In recent decades RNA viruses have been a major cause of emerging and re-emerging infections, including Zika virus (ZIKV), Dengue virus (DENV), Human Immunodeficiency Virus (HIV), Ebola virus (EBOV), yellow fever virus (YFV), and recently, severe acute respiratory syndrome coronavirus 2 (SARS-CoV-2). The resulting epidemics and pandemics have caused high morbidity, mortality, and economic costs ^1^.

To date, our ability to manage these outbreaks is hampered by the challenge of making a definitive clinical diagnosis, as many of these viruses are often clinically indistinguishable from those caused by co-circulating viruses and some bacterial pathogens ^2, 3^. Diagnostic tests can be limited by low specificity, in the case of serological tests, or require *a priori* knowledge of the viruses to be targeted in the case of RT-PCR (reverse transcription-polymerase chain reaction). For these reasons, acute febrile illness often remains undiagnosed, leading to a failure of epidemiological surveillance. Rapid genomic surveillance systems are essential to identify emerging viruses, detect and monitor viral diversity, and be able to prepare for or even prevent new outbreaks ^4^.

New applications have been driven by technological advances in sequencing. The first examples of real-time genomic surveillance ^5, 6^ were conducted using targeted amplicon sequencing on the MinION (Oxford Nanopore Technologies). These studies exploited the portability of nanopore sequencing to achieve a faster turnaround time by sequencing the samples close to where they were collected. While successful for the EBOV epidemic in West Africa, and ZIKV, chikungunya virus (CHIKV), DENV, and YFV outbreaks in Brazil ^7– 9^, this approach is best when the outbreak strain is known, but is less suited to diverse viral groups or virus discovery.

Viral metagenomics, the process of sequencing the total viral nucleic acid content in a sample (typically cDNA or DNA), allows the genomic characterization of known and novel viruses in an untargeted manner. This technique is particularly useful for diagnostic, clinical laboratories, and public health surveillance ^10– 12^. However, viral metagenomic sequencing directly from clinical samples suffers from poor sensitivity, especially in samples with a low abundance of viral genomic material relative to host-derived nucleic acid ^13– 15^. Nanopore metagenomic sequencing has already shown promise by Kafetzopoulou *et al.* (2018) who reported metagenomic sequencing of Lassa virus (LASV), DENV, and CHIKV samples ^16^, and by Lewandowski *et al.* (2019) who sequenced the Influenza virus from respiratory samples ^17^. In both of these studies the approach used was SISPA ^18^ which generates double-tagged cDNA during second-strand synthesis rather than by the SMART mechanism. 

In this study, we describe a high-sensitivity, low input, SMART (Switching Mechanism at the 5′ end of RNA Template) approach for nanopore metagenomics of RNA viruses from isolated samples or from clinical samples. The SMART approach was originally described in 2001 ^19^, using oligo-dT priming to target polyadenylated mRNA molecules. We adapted this method to random priming for cDNA synthesis followed by PCR amplification (SMART-9N), and Rapid SMART-9N barcoded PCR primers are used in the PCR amplification enabling the addition of barcodes in a single step. SMART-9N recovered a high proportion of viral reads from a ZIKV isolate titrated down to 6 FFU/mL of material input, including 94.4% of the genome in a single read. The methods were validated in YFV and SARS-CoV-2 directly from plasma and residual nasopharyngeal samples, respectively. The performance of this assay was compared to a gold-standard multiplex PCR method ^20^, demonstrating improvements in sequencing sensitivity, coverage, depth, cost, and complexity of both SMART-9N and Rapid SMART-9N, enabling enhanced pathogen detection for both diagnosis and surveillance of RNA viruses.

## Methods

### Sample collection

ZIKV isolate strain BeH815744 (GenBank Accession No. KU365780) was propagated into Vero cells (CCL-81; ATTC, Manssas, USA) with minimum essential medium (MEM) for 2 hours at 37°C and 5% CO _2_. The supernatant was removed, and MEM supplemented with 2% fetal bovine serum, 1% penicillin, and 1% streptomycin, to prevent bacterial growth. The cells were incubated for 4 days until 70% of cytopathic effect. Subsequently, the cell culture supernatant was collected and viral replication was confirmed through real-time quantitative reverse transcription-PCR (RT-qPCR) ^21^ and quantified by focus-forming units (FFU) assay in Vero cells ^22^. This sample was used to assess the performance of all three methods: multiplex PCR, SMART-9N, and Rapid SMART-9N. The metagenomic approaches, SMART-9N, and Rapid SMART-9N, were tested in different serial ten-fold MEM dilutions up to 1-1,000,000 to assess the limit of detection ( *Extended data:* Tables S1 and S2).

For methodological validation, human clinical samples included:

- 41 plasma samples previously positive for YFV by RT-qPCR ^23^ collected between January 11 and May 10, 2018, with a ct-value cut-off of ≤ 37 ( *Extended data:* Table S2), obtained from *Hospital das Clínicas*, *Faculdade de Medicina da Universidade de São Paulo* (HC-FMUSP), São Paulo, Brazil. The samples were amplified by multiplex PCR and only those amplified and visible on agarose gel were sequenced. From these, samples with Ct-values between 4.6 and 33 were selected for SMART-9N and Rapid SMART-9N sequencing ( *Extended data:* Table S1);
- Ten residual nasopharyngeal samples previously positive for SARS-CoV-2 by RT-qPCR ^24^, collected between November 17, 2020, and January 05, 2021, with Ct-values ≤ 34, obtained from the *Instituto de Medicina Tropical*, *Faculdade de Medicina da Universidade de São Paulo*, Brazil ( *Extended data:* Table S2). These samples were selected for multiplex PCR and Rapid SMART-9N sequencing ( *Extended data:* Table S1).

Participants or their legal representatives provided signed informed consent. The ethical overview was provided by the institutional review boards at HC-USP and the Infectious Diseases Institute “Emilio Ribas'' for the YFV study as part of the Efficacy of Sofosbuvir as a treatment for Yellow Fever study, protocol number CAAE 82673018.6.1001.0068. For the SARS-CoV-2 study, ethical approval was by the national ethical review board (Comissão Nacional de Ética em Pesquisa), protocol number CAAE 30127020.0.0000.0068. 

### Nucleic acid extraction and RT-qPCR testing

For the ZIKV isolate, the viral RNA was isolated from 200 μl of the culture supernatant material using the QIAamp Viral RNA Mini Kit (Cat No. 52906, Qiagen, Germany) according to the manufacturer's instructions and eluting in 50 μl of elution buffer. For the SARS-CoV-2 nasopharyngeal and YFV plasma samples, 500 μl of the clinical samples were centrifuged for 5 minutes at 10,000 g. The viral RNA was extracted from 200 μl of supernatant material using the NucliSENS EasyMag system (BioMerieux, UK) automated DNA/RNA extraction platform, and eluted in 50 μl. Ct-values were previously determined for all samples by RT-qPCR for ZIKV ^21^, YFV ^23^, and SARS-CoV-2 ^23, 24^.

44 μl of the extracted RNA was treated using TURBO DNase (Cat No. AM2239, Thermo Fisher Scientific, USA) at 37°C for 30 min to remove residual DNA before being cleaned up and concentrated to 10 μl using Zymo RNA clean & concentrator-5 (Cat No. R1016, Zymo Research, USA).

### Multiplex tiling PCR

***cDNA synthesis and Multiplex PCR***

Samples were submitted to whole-genome sequencing using a gold-standard multiplex PCR amplicon sequencing approach ^9, 20^. Briefly, the cDNA was produced from RNA-positive samples using random hexamers (Cat No. N8080127, Thermo Fisher Scientific, USA) and ProtoScript II reverse transcriptase (Cat No. E6560, New England BioLabs, USA) according to the manufacturer's instructions. The cDNA was then amplified with the multiplex PCR assay previously standardized for ZIKV, YFV ^20^, and with the ARTIC V3 multiplexed amplicon scheme for SARS-CoV-2 ^25^ ( https://artic.network/ncov-2019). PCR products were purified using a 1:1 ratio of AMPure XP beads (Cat No. A63881, Beckman Coulter, UK) and quantified using fluorimetry with the Qubit dsDNA High Sensitivity Assay (Cat No. Q32854, Life Technologies, USA) on the Qubit 3.0 instrument (Life Technologies, USA) both according to manufacturer's instructions. A gel was prepared with the PCR products using the E-gel Agarose 2% (Cat No. G402002, Thermo Fisher Scientific, USA) on E-gel equipment (Thermo Fisher Scientific, USA) and the run was performed until the bands were distinguishable by transillumination. The samples that presented bands with the expected amplicon size (approximately 500 base-pairs (bp)) were selected for MinION sequencing. 

***Nanopore library preparation and sequencing***

MinION libraries were prepared using a total input of 100 ng, barcoded, and pooled in an equimolar fashion using the EXP-NBD104 (1-12), and EXP-NBD114 (13-24) Native Barcoding Kits (ONT, UK). Sequencing libraries were generated using the SQK-LSK109 Kit (ONT, UK). 20 ng of the final libraries were loaded onto FLO-MIN106 flow cells on the MinION device (ONT, UK) and sequenced using MinKNOW 1.15.1 with the standard 48-hour run script.

### SMART-9N

***SMART cDNA synthesis and PCR***

For the SMART-9N cDNA synthesis, 10 μl of the concentrated RNA, 1 μl NEB bRT 9N (AAGCAGTGGTATCAACGCAGAGTACNNNNNNNNN, 2μM) and 1 μl 10 mM deoxyribonucleotide triphosphate (dNTPs) mix (Cat No. N0447L, New England Biolabs, USA) were mixed and incubated for 5 min at 65°C, then cooled on ice. 4 μl SuperScript IV first-strand buffer, 1 μL 0.1 M DTT, 1 μl RNase OUT, 1 μl NEB SSP (RNA oligo) (GCTAATCATTGCAAGCAGTGGTATCAACGCAGAGTACATrGrGrG, 2 μM), and 1 μL SuperScript IV (Cat No. 18091200, Thermo Fisher Scientific, USA) were mixed with the 12 μl annealed RNA before incubation for 90 min at 42°C followed by 10 min at 70°C. These double-tagged cDNA products were amplified using 5 μl Q5 reaction buffer (Cat No. M0491, New England BioLabs, USA), 0.5 μl 10 μM dNTP, 1 μl NEB PCR (AAGCAGTGGTATCAACGCAGAGT, 20 μM), 15.75 μl Nuclease-free water (NFW), 0.25 μl Q5 DNA polymerase, and 2.5 μl of cDNA. PCR cycling conditions were: 98°C for 45 sec, followed by 30 cycles of 98°C for 15 sec, 62°C for 15 sec, and 65°C for 5 min and a final step of 65°C for 10 min. The products were purified using a 1:1 ratio of AMPure XP beads (Cat No. A63881, Beckman Coulter, UK) and quantified using fluorometry with the Qubit dsDNA High Sensitivity assay (Cat No. Q32854, Life Technologies, USA) on the Qubit 3.0 instrument (Life Technologies, USA) both according to manufacturer's instructions.

***Nanopore library preparation and sequencing***

MinION libraries were prepared using 50 ng of each amplified cDNA, barcoded, and pooled in an equimolar fashion using the EXP-NBD104 (1-12) and EXP-NBD114 (13-24) Native Barcoding Kits (ONT, UK). Sequencing libraries were generated using the SQK-LSK109 Kit. 50 ng of the final libraries were loaded onto FLO-MIN106 flow cells on the MinION device (ONT, UK) and sequenced using MinKNOW 1.15.1 with the standard 48-hour run script.

### Rapid SMART-9N

***SMART cDNA synthesis and barcoded PCR***

For the rapid SMART-9N cDNA synthesis, 10 μl of the concentrated RNA, 1 μl RLB RT 9N (TTTTTCGTGCGCCGCTTCAACNNNNNNNNN, 2 μM) and 1 μl 10 mM dNTPs (Cat No. N0447L, New England BioLabs, USA) were mixed and incubated for 5 min at 65°C, then cooled on ice. 4 μl SuperScript IV First-strand Buffer, 1 μL 0.1 M DTT, 1 μl RNase OUT, 1 μl RLB TSO (RNA oligo) (GCTAATCATTGCTTTTTCGTGCGCCGCTTCAACATrGrGrG, 2 μM), and 1 μL SuperScript IV (Cat No. 18091200, Thermo Fisher Scientific, USA) was mixed with the 12 μl annealed RNA before incubation for 90 min at 42°C followed by 10 min at 70°C. These double-tagged cDNA products were amplified using 25 μl LongAmp Taq 2X master mix (Cat No. M0287, New England BioLabs, USA), 19.5 μl of NFW, 0.5 μl RLB (1-12) from SQK-RPB004 kit (ONT, UK) and 5 μl of cDNA. PCR cycling conditions were: 95°C for 45 sec, followed by 25–30 cycles of 95°C for 15 sec, 56°C for 15 sec, and 65°C for 5 min and a final step of 65°C for 10 min. For the PCR step, the RLB PCR (TTTTTCGTGCGCCGCTTCA, 20 μM) can be used as PCR control, changing the cycles conditions to 98°C for 45 sec, followed by 25–30 cycles of 98°C for 15 sec, 62°C for 15 sec, and 65°C for 5 min and a final step of 65°C for 10 min. The products were purified using a 1:1 ratio of AMPure XP beads (Cat No. A63881, Beckman Coulter, UK), quantified using fluorimetry with the Qubit dsDNA High Sensitivity Assay (Cat No. Q32854, Life Technologies, USA) on the Qubit 3.0 instrument (Life Technologies, USA) both according to manufacturer's instructions.

***Nanopore library preparation and sequencing***

MinION libraries were prepared using 200 Femtomolar (fM) in 10 µL of 10 millimolar (mM) Tris-HCl pH 8.0 with 50 millimolar (mM) NaCl. 1 µl RAP adapter was added to the library and incubated at room temperature for 5 min. The final libraries were loaded onto FLO-MIN106 flow cells on the MinION device (ONT, UK) and sequenced using MinKNOW 1.15.1 with the standard 48-hour run script.

### Bioinformatics workflow

Raw FAST5 files were basecalled using Guppy software version 2.2.7 GPU basecaller (Oxford Nanopore Technologies), then demultiplexed and trimmed using Porechop v.0.3.2pre. The barcoded FASTQ files were aligned and mapped to the reference genome (GenBank accession no. JF912190 (YFV), KX893855.1 (ZIKV), and MN908947 (SARS-CoV-2)) using minimap2 version 2.28.0 ^26^ and converted to a sorted BAM file using SAMtools ^27^. NanoStat version 1.1.2 ^28^ was used to compute the number of raw reads and minimum contig length to cover 50 percent of the genome (N50) of the aligned reads. Tablet 1.19.05.28 ^29^ was used for genome visualization, and to compute the number of mapped reads, percentage of genome coverage, and coverage depth. Samtools stats and samtools depth ^27^ were used to calculate longest reads and genome coverage at 20x respectively. For the multiplex PCR analysis, length filtering, quality test, and primmer trimming were performed for each barcode using artic guppyplex and variant calling and consensus sequences using artic minion Nanopolish version from ARTIC bioinformatics pipeline. For the SMART-9N and Rapid SMART-9N, called variants were detected with medaka_variants and the consensus sequence was built using medaka_consensus (ONT, UK).

For detection of other viral RNA in the clinical samples, taxonomic classification was conducted using Kraken version 2.0.8-beta, using the MiniKraken2_v1_8GB Kraken 2 Database, which comprises eukaryotic, bacterial, viral, and archaeal Refseq complete genomes. After classification, those classified as “Viruses” in the output reports, were analysed for each barcode individually. The manual downstream analyses consisted on mapping each FASTQ file to the respective potential FASTA of the virus of interest downloaded from NCBI. Tablet ^29^ was used to verify the genomes mapping pattern, and to exclude the possibility of genome chimera or false positive interpretation. A dsDNA virus genus Pa6virus, family Siphoviridae was identified in one YFV sample, and the pipeline described above was used to generate consensus sequences, using the reference sequence (NC_018838.1).

## Results

**Figure 1.**
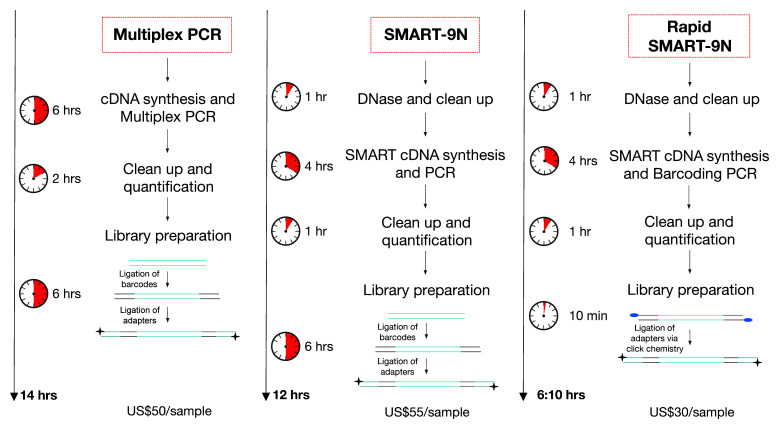
Comparison between the steps and cost of the workflows – tiling amplicon sequencing – Multiplex PCR, SMART-9N, and Rapid SMART-9N. Abbreviations: *SSP*: Strand Switching Primer. US$, American dollar.

In this study, we designed two methodologies, SMART-9N and Rapid SMART-9N. The SMART-9N approach is based on the NEBNext Single-cell/low-input RNA (cat no. E6420, New England BioLabs, USA) modified to use random priming and native barcoding library preparation (cat no. SQK-NBD104, ONT, UK). The NEB method uses single-primer PCR amplification which we found we could perform using barcoded primers from the Rapid PCR Barcoding Kit (SQK-RBP004, ONT, UK) if we modified the sequence of the RT and SSP oligos. This approach allows for amplification of RNA in the picogram input range (data not shown) making it ideal for low-input applications. We compared the complexity, costs, and time required of laboratory work to a previously standardized multiplex tilling PCR approach ^20^. Compared to multiplex PCR, the total time of hands-on laboratory work dropped 15% and 57% for the SMART-9N and Rapid SMART-9N respectively, and reagent costs were reduced by 40% and 45% ( Figure 1).

### Multiplex PCR sequencing of ZIKV isolate and YFV and SARS-CoV-2 clinical samples

**Figure 2.**
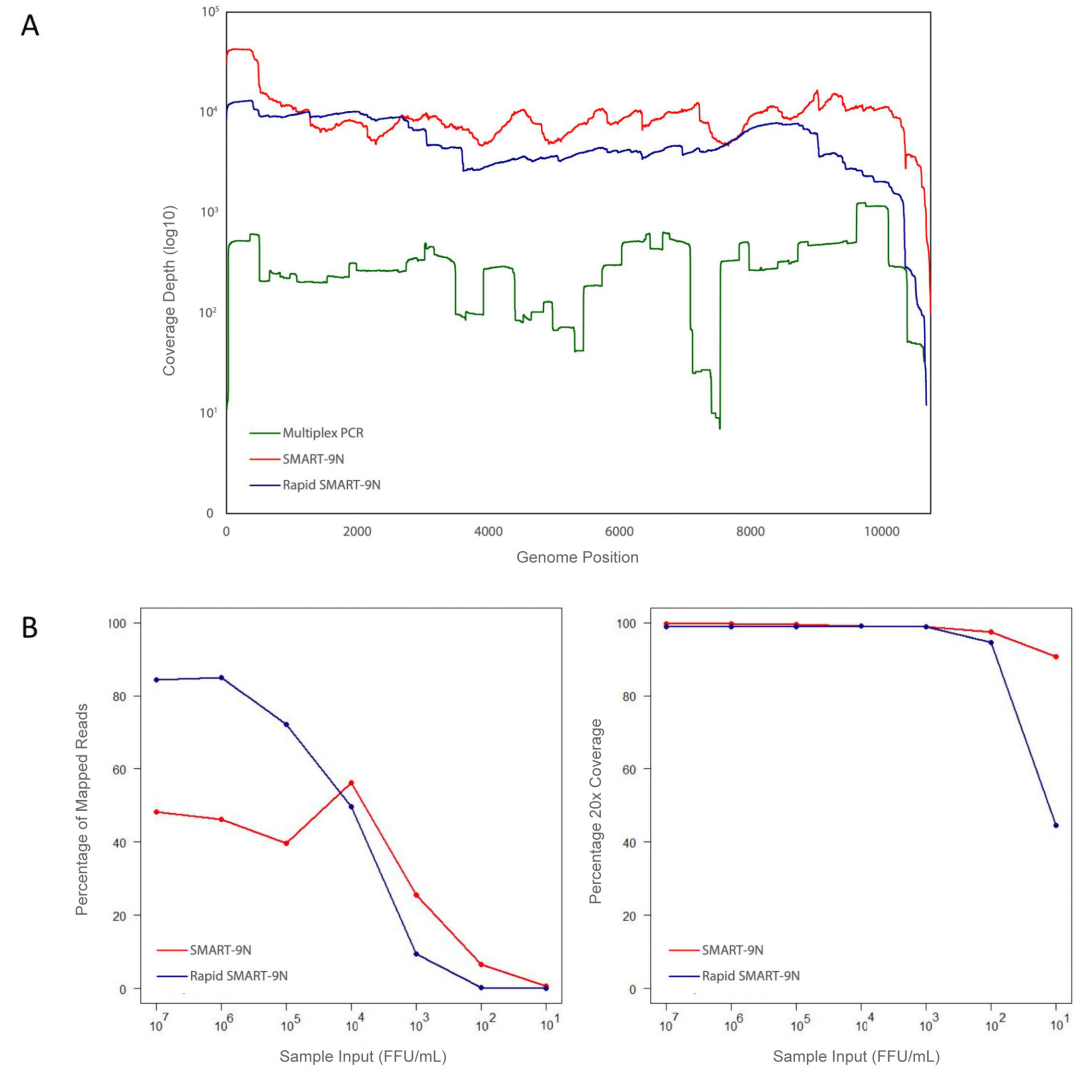
Comparison of multiplex PCR, SMART-9N, and Rapid SMART-9N approaches. **A**) Genome coverage of ZIKV genome for reference material as to coverage of reads mapped to the genome reference position comparing the multiplex PCR, SMART-9N, and Rapid SMART-9N approaches. **B**) Limit of detection of the SMART-9N and Rapid SMART-9N methods analyzing the proportions of reads mapping to the appropriate reference viral sequence across a range of sample input (FFU/mL) on the left plot and percentage of the reference genome sequenced to a minimum depth of 20-fold in the data generated across a range of sample input (FFU/mL) on the right plot.

Initial testing was performed on a serial dilution of ZIKV isolate, which was subjected to the gold-standard multiplex PCR approach followed by MinION sequencing ^20^. This sample had a Ct-value of 15.1 and an RNA titer of 6e7 FFU/mL ( *Extended data:* Table S2). The percentage of mapped reads was 55.99% with an average depth of 326.98x covering 98.74% of the viral genome covered with at least 1 read ( *Extended data:* Table S3; Figure 2).

**Figure 3.**
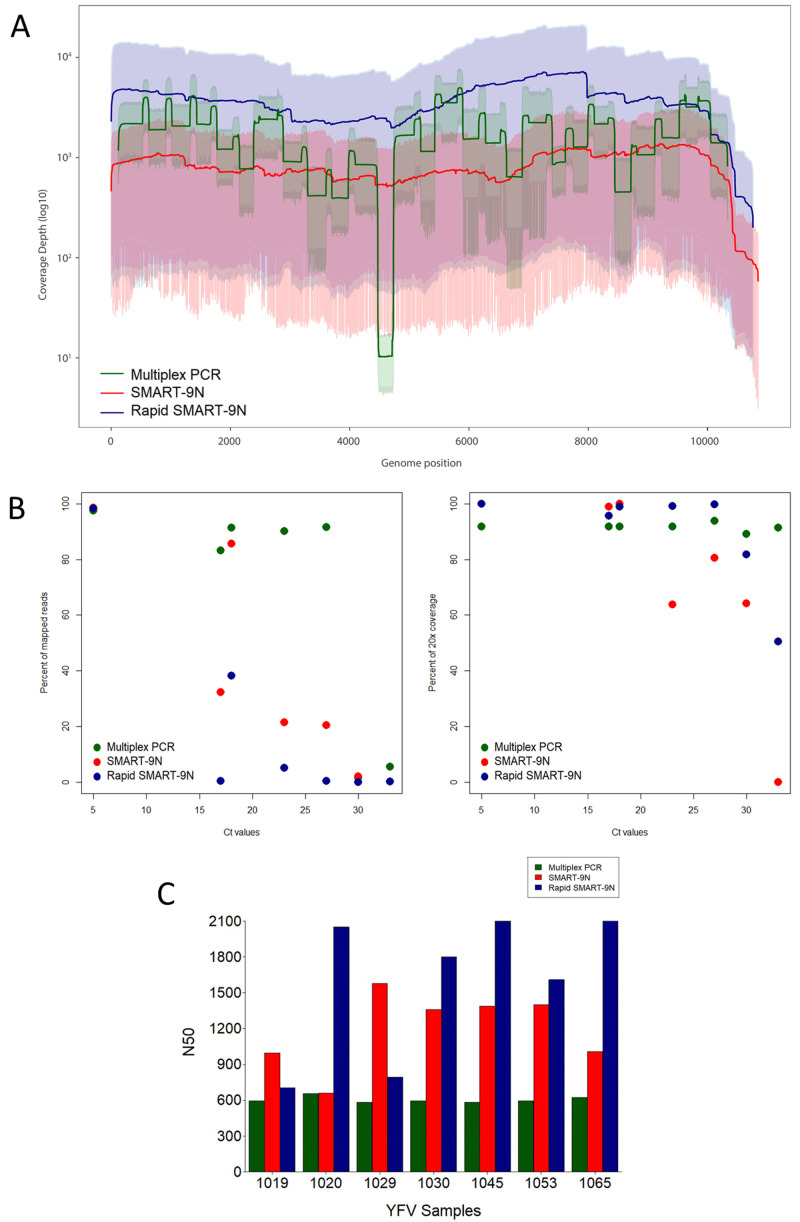
Comparison of multiplex PCR, SMART-9N, and Rapid SMART-9N results for YFV clinical samples. **A**) Average genome coverage depth and 95% of reads mapped to the genome reference position. **B**) Proportion of reads mapping to the reference genome across a range of Ct *-*values (left) and percentage of the reference genome sequenced to a minimum depth of 20-fold across a range of Ct *-*values (right). **C**) N50 of each sample in bp. (n=7 samples).

The assay was also performed on 41 human clinical samples positive for YFV RNA by RT-qPCR from the 2018 YFV epidemic in São Paulo, Brazil. The median Ct-value was 27.74, ranging from 4.6 to 37 corresponding to 1 to 1.5e10 genome copies per mL of plasma ^30^. After PCR product quantification and the E-gel agarose gel run, 21 samples presented specific bands distinguishable by transillumination and were selected to continue nanopore library preparation and sequencing ( *Extended data:* Table S3). The sequenced YFV samples (n=21) had a median Ct-values = 25.57, between 5 and 37 generated in one barcoded ONT library. The percentage of mapped reads ranged from 1.71% to 97.47%, with an average depth between 72.5x to 3370x, and the majority samples with genome coverage around 99.82% being the lowest 78.11% ( *Extended data:* Table S3; Figure 3). Genome regions with a depth of <20x coverage were represented with N characters.

**Figure 4.**
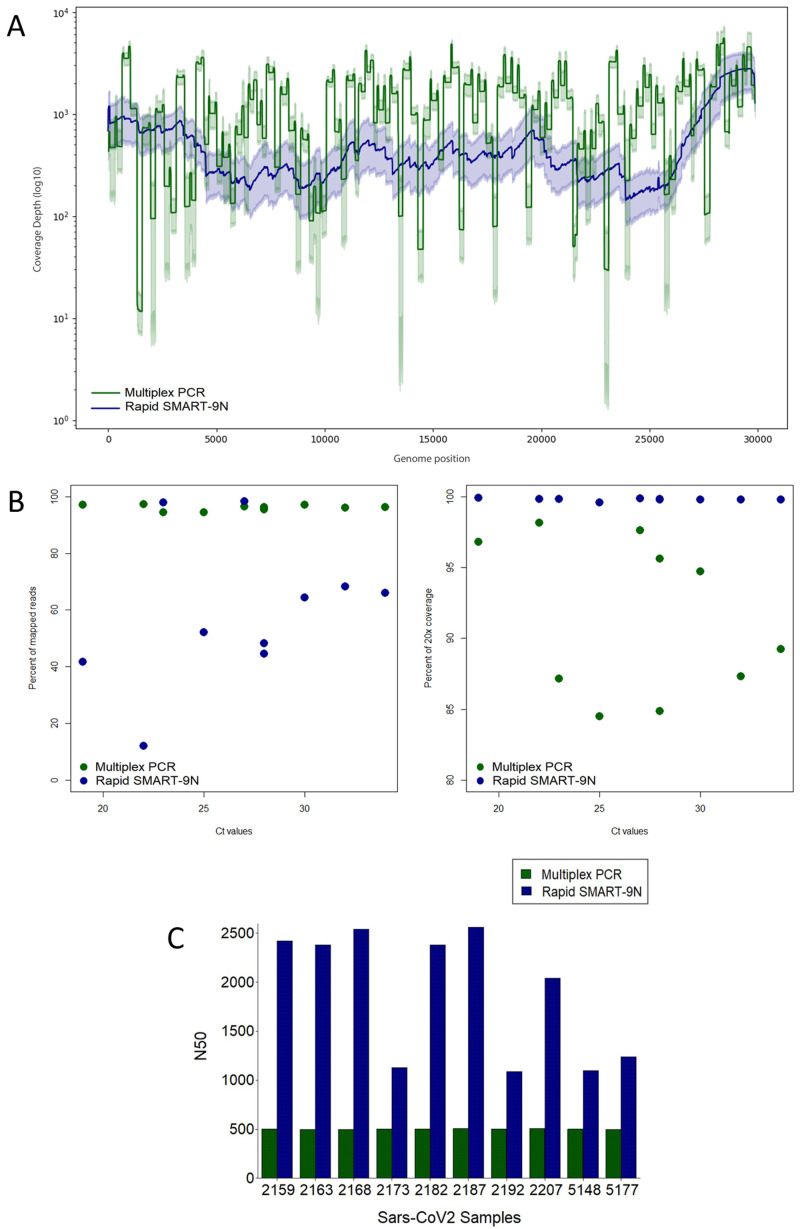
Comparison of multiplex PCR, and Rapid SMART-9N results for SARS-CoV-2 clinical samples. **A**) Average genome coverage depth and 95% of reads mapped to the genome reference position. **B**) Proportion of reads mapping to the reference genome across a range of Ct *-*values (left) and percentage of the reference genome sequenced to a minimum depth of 20-fold across a range of Ct *-*values (right). **C**) N50 of each sample in bp. (n=10 samples).

For SARS-CoV-2 the assay was performed in 10 residual nasopharyngeal samples positive for SARS-CoV-2 RNA by RT-qPCR in April 2020 in São Paulo, Brazil. The median Ct-value was 26.9, ranging from 21.8 to 33.3 corresponding to 1.3e2 to 2.4e5 genome copies per mL. The percentage of mapped reads ranged from 94.51% to 97.27%, with an average depth of 821.77x to 1570x, and genome coverage median of 98.8%, ranging from 95.90% to 99.92% ( *Extended data:* Table S3; Figure 4).

### SMART-9N and Rapid SMART-9N of ZIKV isolated-culture samples and limit of detection

For ZIKV, the titrated isolate RNA was diluted with serial ten-fold dilutions, up to 1:1,000,000 corresponding to 6e7 to 6 FFU/mL, and subjected to SMART-9N ( *Extended data:* Table S4). The test resulted in a median of 99.7% genomic coverage for the tested dilutions with the lowest 99.02% for the 1:1,000,000 dilution. The percentage of genome coverage at 20x was 90.7% with 6 FFU/mL up to 99.73% with 6e7 FFU/mL of material input. The coverage depth was up to 10010x, and with 6 FFU/mL of material was 154.25x, compatible with single-cell assays. The average of mapped reads ranged from 56.29% to 0.52%. The median N50 was 1.7kb and when the reads were individually analysed, the test obtained complete ZIKV genome coverage in a single read (approximately 11kb longest read) ( Figure 2).

The same 1:1,000,000 dilution was used to test the Rapid SMART-9N approach. The lowest proportions of mapped reads observed were 0.06% and the highest 86.15%. The majority of samples returned a percentage of 99.87%, with 87.58% for the 6 FFU/mL dilution test. The median of the percentage of genome coverage at 20x was 90.73% and the N50 was 2.27kb ( Figure 2). 

The method was performed using 1 μl and 0.5 μl RLB barcodes from the SQK-RPB004 kit (ONT) with 6e7 FFU/mL of material input. The test resulted in 99.7% genomic coverage for both 1μl and 0.5 μl, and N50 of 1.84kb and 2.11kb respectively ( *Extended data:* Table S5). 

### SMART-9N and Rapid SMART-9N of YFV clinical samples

After validating the methods on ZIKV isolate we next applied them to clinical samples. Starting with the SMART-9N, seven representative human clinical samples positive for YFV RNA, already sequenced with the multiplex PCR method, with Ct-values between 4.6 and 33 were selected ( *Extended data:* Table S6). A total of 86% of the samples presented genome coverage greater than 99.9% ranging from 95.11% to 99.99% with Ct-values of 33 and 18 respectively, and a minimum average depth of 3.2x, and a maximum of 3480x ( Figure 3A). The same samples were selected and subjected to the Rapid SMART-9N method ( *Extended data:* Table S7). The highest mapped read percentages observed were 98.26% and 38.18% for Ct-values 4.6 and 17.4, respectively. A total of 86% of the samples presented genome coverage greater than 99.9% with the lowest of 94.28% with a Ct of 33, and the average depth ranged from 21.44x to 2530x ( Figure 3A). We compared the coverage depth with different Ct-values samples across the relevant genome for each method (multiplex PCR, SMART-9N, and Rapid SMART-9N) ( *Extended data:* Figure S1). The average coverage depth revealed higher genome depth and better coverage pattern across the genome for the metagenomics methods when compared to the targeted multiplex PCR method.

All the seven sequenced samples with both methods were compared to the multiplex results. Despite the decrease in the proportion of mapped viral reads across the range of Ct-values ( Figure 3B) with the SMART-9N and Rapid SMART-9N, we could obtain a comparable correlation (SMART-9N R=0.91, p=0.005; Rapid SMART-Metagenomics R=-0.86, p=0.012,). The correlation showed a decreased proportion of viral reads as the Ct-values increased, with a considerable level of variation (0.3% to 98.6% with SMART-9N and 0.16% to 98.26% with Rapid SMART-9N method) between samples and methods ( *Extended data:* Tables S6 and S7).

20-fold genome coverage across the Ct-values was compared between all methods, presented in Figure 3B. In the multiplex approach, the average of the genome coverage was 78.9% with a minimum of 35.01% for Ct 33 compared to 71.5% and 89.3% for SMART-9N and Rapid SMART-9N with a minimum of 0% and 50.5%, respectively ( *Extended data:* Tables S6 and S7).

For this subset of samples, we also compared the N50 results from the approaches for each sample ( Figure 3C). Here we found the range was 525bp to 660bp for multiplex PCR, 659bp to 1.58kbp for SMART-9N, 705bp to 2.16kb for Rapid SMART-9N. The median was 599.8bp, 1.6kbp, and 1.2kbp for the multiplex PCR, SMART-9N, and Rapid SMART-9N respectively ( *Extended data:* Tables S6 and S7). For the YFV clinical samples, the longest reads observed were 10.08kb and 9.12kb for the SMART-9N, and Rapid SMART-9N, respectively 93.33% and 84.44% of the entire viral genome.

### Rapid SMART-9N of SARS-CoV-2 clinical samples

SARS-CoV-2 clinical samples were subjected to the Rapid SMART-9N approach. Due to the emergence of SARS-CoV-2 during the validation of the protocols, we chose to test for SARS-CoV-2 only with the Rapid SMART-9N protocol, for being a faster and promising technique to be used in the course of the pandemic.

Reads mapping to reference virus genome (isolate: Wuhan-Hu-1, GenBank Accession No. MN908947) were present in all ten samples up to Ct-value 34 (total reads ranged from 6480 to 93,570 reads). The sequenced samples were compared to the multiplex results and did not show a significant correlation (R=0.49, p=0.15) between the proportion of viral reads with increasing Ct-value (12.15 - 98.22%) ( *Extended data:* Table S8). The genome coverage was 100% in all 10 samples and the lowest coverage depth of 97.51x ( Figure 4A). When comparing each coverage depth across different Ct-values samples for the multiplex PCR, and Rapid SMART-9n methods ( *Extended data:* Figure S2), we could observe a concordant coverage depth and coverage pattern across the genome for both methods.

Comparison of genome coverage 20-fold between multiplex PCR and Rapid SMART-9N across the viral titer range is shown in Figure 4B. The median revealed for the multiplex PCR reactions was 91.59%, minimum 84.49%, and the Rapid SMART-9N 99.79%, minimum of 99.57%. A comparison of the N50 in all the 10 samples was made resulting in a higher N50 of all samples to the Rapid SMART-9N approach, up to 2.56kb. The longest read was 18.48kb, the longest read obtained in this study, comprising approximately 62% of the SARS-CoV-2 genome (29,903 bp) ( Figure 4C).

### Detection of other RNA viruses in clinical samples and Kraken classification

To test the ability of our methods to detect other viruses in our samples, we assessed the taxonomic classification of reads using Kraken for all clinical samples. This allowed for the identification of a dsDNA virus genus Pa6virus, family *Siphoviridae* present in one YFV sample. After identification, the reads were mapped to the reference sequence (NC_018838.1) obtaining 197 reads of the virus, 84.78% of genome coverage with a maximum coverage depth of 32x, and identity of 81.4%. The consensus sequence was generated and bases with a depth of <10-fold were represented with N characters (github.com/CADDE-CENTRE/Rapid-RNA-SMART-Metagenomics). The proportion of unclassified, eukaryota, bacteria, archaea, and viruses reads, for each sample can be found in the *Extended data,* Table S9.

## Discussion

A rapid, sensitive sequencing method for viral metagenomics is key to be able to identify the cause of unknown infections. Although PCR-based testing and amplicon-based sequencing methodologies are available and are very sensitive, they are not suitable for the initial detection of emerging or re-emerging viruses due to the need for gene-specific primers/probes for diagnostic assays or primer panels ^15^. The etiology of suspected infections in acute illness often remains undiagnosed. An untargeted sequencing method remains the best strategy for the identification of unknown viral infections, and the genome sequences provide information about the evolutionary history ^31^, strain identification ^32, 33^, and biology of new pathogens ^14^. This is evidenced by the recent rapid and impactful metagenomic analysis of SARS-CoV-2 early in the pandemic ^34, 35^. 

In this study, we developed two viral metagenomic approaches, SMART-9N and Rapid SMART-9N as non-targeted metagenomics methods for detection and characterization of viral RNA. The two techniques demonstrated excellent specificity (100%) when tested in isolated and clinical samples that had been compared to a gold-standard multiplex PCR method ^5– 9^.

For ZIKV isolated-culture, it was possible to obtain 99.02% of genome coverage with an input of 6 FFU/mL, an amount comparable to other single-cell methods available ^36, 37^ for the SMART-9N approach. For the Rapid SMART-9N, 87.58% of the ZIKV genome was recovered for the same dilution of 1:1,000,000. The sensitivity and high yield of viral sequences from clinical YFV and SARS-CoV-2 samples make it potentially feasible to directly perform metagenomic MinION whole-genome sequencing, even for higher Ct-values. Representative clinical samples with Ct-values between 4.6 and 33 for YFV, and between 21.8 and 33.3 for SARS-CoV-2 were selected to test the genome recovery for the viruses tested. Notably, the SMART-9N and Rapid SMART-9N methods were effective in directly genome sequencing clinical samples for both viruses tested since viral reads were detected in all samples, until in samples with 1 genome copy per mL.

Evaluating the read length during the validation, we observed that our approach generated very long reads when compared to other metagenomics approaches ^16, 17^. In this study, we generated the whole ZIKV and YFV genome and approximately 60% of the SARS-CoV-2 genome in one single read. The N50 of the methods was up to 2.91kb with the isolated samples and up to 2.56kb with SARS-CoV-2 clinical samples and the Rapid SMART-9N approach. The average N50 for the clinical samples using the SMART-9N was 1.2kb and for Rapid SMART-9N was 1.6kb, a difference we believe can be explained by the different tag sequence. When a single PCR primer is used any templates that self-anneal will not amplify resulting in an enrichment of longer products for SMART-9N. When looking at the average coverage depth and the CI of the metagenomics methods, we observe consistent amplification across the entire genome. Increased N50 provides higher confidence in individual read taxonomic assignment, improves mapping confidence, *de novo* assembly, and the ability to detect viral recombinations ^38, 39^. To our knowledge 18.5kb is the longest viral cDNA published to date produced by the Rapid SMART-9N method, this was likely due to the fact that LongAmp polymerase is used for barcoded primers as per ONT recommendations whereas Q5 polymerase was used for SMART-9N.

We also compared the complexity, costs, and time required of laboratory work to the multiplex tilling PCR approach ^20^. Using this approach, our Rapid SMART-9N reduced the complexity, time, and cost from sample to sequence. The addition of the barcode during PCR decreased the library preparation time from 6 hours to 10 minutes, reducing the cost due to no longer needing enzymes for end-preparation and ligation which also rely on a cold chain making them inconvenient to use in the field. The total time of laboratory work dropped 15% and 57% for the SMART-9N and Rapid SMART-9N respectively when compared to the multiplex PCR. The costs when using Rapid SMART-9N dropped 45% and 40% compared to SMART-9N and multiplex PCR respectively. Using half of the volume for the rapid barcode primers doubles the number of samples that can be processed with the kit from 72 to 144. This protocol has the potential to be further optimized and used in a lyophilized formulation with the elimination of any cold chain. These results demonstrate that the Rapid SMART-9N is an important approach in both the laboratory or field settings.

The YFV and SARS-CoV-2 clinical samples were also analysed in an untargeted way, mapped to an available reference database and analysed manually in order to screen potential microbial contamination and/or co-infections. The methods allowed the identification of an unknown co-infection in a YFV clinical sample, a dsDNA virus genus Pa6virus, family Siphoviridae, had the full genome characterized. We showed that our non-targeted sequencing approach offers an opportunity for simultaneous testing for a wide range of potential pathogens, providing a faster route to identification followed by a potential specific treatment.

### Limitations of the method

The overwhelming majority of reads are derived from the human host, mainly in clinical samples with high Ct-values (with a low relative abundance of viral genomic material) or in samples with degraded genetic material due to poor storage conditions. This is a limiting factor for the sensitivity of the approach that could result in low or no coverage of the infectious agent. While the DNase treatment dramatically improves sensitivity, more work is needed in depleting highly abundant rRNA species which are recovered as a result of the random priming. Lower sensitivity is seen in our study when comparing the number of viral reads from the ZIKV isolates to the YFV and SARS-CoV-2 clinical samples. The reduction in the number of viral reads as the Ct-value increases is due to the total level of non-viral host/background nucleic acid present and provides an upper limit for the approach above which amplicon sequencing is more useful. The difference we observed in N50 between SMART-9N and Rapid SMART-9N cannot be easily resolved so we recommend using SMART-9N for best representation and Rapid SMART-9N when speed is more important.

## Conclusion

Here we demonstrate a sensitivity workflow across viral isolate and clinical samples which takes advantage of long-read nanopore sequencing technology by generating long (up to 18.5 kb) cDNA amplification products for viral metagenomics. Therefore, our metagenomic sequencing approaches offer an opportunity for sensitive identification and characterization of RNA viruses directly from isolates or clinical samples with a range of viral loads. Also, the Rapid SMART-9N demonstrated a simple, low-cost, and faster method, promising for routine use in the research laboratory as well as in the field.

## Data Availability

All raw files with the host reads depleted, and consensus sequences generated in this study can be found at https://github.com/CADDE-CENTRE/Rapid-RNA-SMART-Metagenomics Repository: CADDE-CENTRE/Rapid-RNA-SMART-Metagenomics, DOI: 10.5281/zenodo.5391968. License CC0. This project contains the following underlying data: SARS_CoV_2_CONSENSUS_SEQUENCES (SARS-CoV-2 consensus sequences (n=10) generated by multiplex PCR and Rapid SMART-9N methods). YFV_CONSENSUS_SEQUENCES (YFV consensus sequences (n=7) generated by multiplex PCR, SMART-9N, and Rapid SMART-9N methods). ZIKV_CONSENSUS_SEQUENCES (ZIKV reference consensus sequences (n=1) generated by multiplex PCR, SMART-9N, and Rapid SMART-9N methods). ZIKV_Multiplex_PCR_RAW_FILES (Raw data (fastq) of ZIKV, SARS-CoV-2, and YFV generated in this study). Repository: CADDE-CENTRE/Rapid-RNA-SMART-Metagenomics/Supplementary_material_SMART_9N.pdf, DOI: 10.5281/zenodo.5391968. License CC0. This project contains the following extended data: Table S1 - Description of samples collected and protocol realized to each sample. Table S2 - Description of samples positive for Zika virus (ZIKV) reference sample strain BeH815744 (n=1), yellow fever virus (YFV) (n=41), and severe acute respiratory syndrome coronavirus 2 (SARS-CoV-2) (n=10) by real-time quantitative reverse transcription PCR with the corresponding sample types, Ct-values, estimated focus forming units (FFU) per milliliter or estimated genome copies per mL, and the virus reference size (nts). Table S3 - Summary of virus nanopore sequencing data using the tiling multiplex PCR approach of Zika virus reference strain BeH815744 (ZIKV) (n=1), yellow fever virus (YFV) (n=21), and severe acute respiratory syndrome coronavirus 2 (SARS-CoV-2) (n=10) samples with the corresponding Ct-values. Table S4 - Sequencing summary and alignment statistics results for Zika vírus (ZIKV) reference sample strain BeH815744 using the SMART-9N method during development (n = 1 sample) according to the material input (FFU/mL). Table S5 - Sequencing summary and alignment statistics results for Zika virus reference sample strain BeH815744 using the Rapid SMART-9N method during development (n = 1 sample) according to the material input (FFU/mL). Table S6 - Sequencing summary and alignment statistics results for yellow fever virus (YFV) plasma samples (n=7) using the SMART-9N protocol during method validation according to the Ct-values. Table S7 - Sequencing summary and alignment statistics results for yellow fever virus (YFV) plasma samples (n=7) using the Rapid SMART-9N protocol during method validation according to the Ct-values. Table S8- Sequencing summary and alignment statistics results for Severe acute respiratory syndrome coronavirus 2 (SARS-CoV-2) clinical samples (n=10) using the Rapid SMART-9N protocol during method validation according to the Ct-values. Table S9 - Proportion in the percentage of unclassified, Eukaryota, bacteria, archaea, and viruses reads, for each sample according to the Kraken classification distribution and metagenomics methodologies. Figure S1 - Comparison of genome coverage depth across the yellow fever virus (YFV) genome for different methods (i.e., multiplex PCR, SMART-9N, and Rapid SMART-9N) in all clinical samples tested with different Ct-values. YFV (n=7) Figure S2 - Comparison of genome coverage depth across the Severe acute respiratory syndrome coronavirus 2 (SARS-CoV-2) virus genome for different methods (i.e., multiplex PCR, and Rapid SMART-9N) in all clinical samples tested with different Ct-values. SARS-CoV-2 (n=10).
