# Supplementary material for: Rapid viral metagenomics using SMART-9N amplification and nanopore sequencing
Source: Wellcome Open Res. 2023 Apr 24;6:241. Originally published 2021 Sep 20. [Version 2] doi: 10.12688/wellcomeopenres.17170.2 (PMC10189296; doi:10.12688/wellcomeopenres.17170.2)
Supplement: Peer review history

# Peer review: Reviewer response for version 2

**DOI:** 10.21956/wellcomeopenres.21114.r56455

**Published:** 2023-05-16

**Authors:** Edward Cunningham-Oakes

**Affiliations:** 1 University of Liverpool, Liverpool, UK

The authors have responded to all revisions, and I have no further comments to make.

Is the rationale for developing the new method (or application) clearly explained?

Yes

Is the description of the method technically sound?

Yes

Are the conclusions about the method and its performance adequately supported by the findings presented in the article?

Yes

If any results are presented, are all the source data underlying the results available to ensure full reproducibility?

Partly

Are sufficient details provided to allow replication of the method development and its use by others?

Yes

Reviewer Expertise:

Metagenomics, microbial genomics, bioinformatics, pathogen detection

I confirm that I have read this submission and believe that I have an appropriate level of expertise to confirm that it is of an acceptable scientific standard.

# Peer review: Reviewer response for version 2

**DOI:** 10.21956/wellcomeopenres.21114.r56454

**Published:** 2023-05-04

**Authors:** Mirela D'arc Ferreira da Costa

**Affiliations:** 1 Instituto de Biologia, Departamento de Genética, Laboratório de Diversidade e Doenças Virais, Federal University of Rio de Janeiro, Rio de Janeiro, Brazil

I confirm that the authors have reviewed all critical points cited in the first version. I have only two small suggestions to improve paper quality: 1. Standardize the way to indicate the FFU/mL (ex. 6X10⁷ instead of 6e7) everywhere (main text and tables); and 2. Several numbers in the Supplementary Material have the decimal place separated by commas and It is important to specify that “N50” and “Longest read” are in kilobases (kbs). Therefore, I believe the article is now refined and accurate enough for wide release. Finally, it is important to emphasize that the article makes an important contribution to the field of metagenomics.

Is the rationale for developing the new method (or application) clearly explained?

Yes

Is the description of the method technically sound?

Yes

Are the conclusions about the method and its performance adequately supported by the findings presented in the article?

Yes

If any results are presented, are all the source data underlying the results available to ensure full reproducibility?

Yes

Are sufficient details provided to allow replication of the method development and its use by others?

Partly

Reviewer Expertise:

Molecular biology, viral metagenomics and bioinformatics.

I confirm that I have read this submission and believe that I have an appropriate level of expertise to confirm that it is of an acceptable scientific standard.

# Peer review: Reviewer response for version 1

**DOI:** 10.21956/wellcomeopenres.18969.r52412

**Published:** 2022-10-03

**Authors:** Mirela D'arc Ferreira da Costa

**Affiliations:** 1 Instituto de Biologia, Departamento de Genética, Laboratório de Diversidade e Doenças Virais, Federal University of Rio de Janeiro, Rio de Janeiro, Brazil

The authors describes a improvement of the SMART approach published in 2001. They developed a rapid viral metagenomics test using initially a random priming for cDNA synthesis and then the SMART-9N barcoded amplification. I believe that it is a important work and should be indexed after a good grammar and concept revision. Some points that should be verified are listed below.

1. In “Introduction” section, it is not clear if the “random priming for cDNA synthesis followed by PCR amplification” refers to the SMART-9N methodology.
2. The ethical approval number for the YFV study is absent in the mean text.
3. The reagents concentration should be more clear in all steps. It is very important to allow replication of the method by others.
4. Some decimal values are incorrectly separated by commas (for example, 15,75 μl Nuclease-free water).
5. In “Bioinformatics workflow” section, it is necessary to introduce the citation of all softwares/tools used (for example, minimap2). Also, the duplicated brackets after SARS-CoV-2 should be removed.
6. It is not clear why the “Clean up and quantification” step has a dramatic time reduction, specially between SMART-9N and Rapid SMART-9N methodologies.
7. In “Rapid SMART-9N of SARS-CoV-2 clinical samples” section, the phrase “were present in all ten seven samples” is not understandable.
8. It is not clear why the SMART-9N approach was not performed to the SARS-CoV-2 clinical samples.
9. The specific viral results obtained by Kraken classification should also be addressed in the Discussion/Limitations section.

Is the rationale for developing the new method (or application) clearly explained?

Yes

Is the description of the method technically sound?

Yes

Are the conclusions about the method and its performance adequately supported by the findings presented in the article?

Yes

If any results are presented, are all the source data underlying the results available to ensure full reproducibility?

Yes

Are sufficient details provided to allow replication of the method development and its use by others?

Partly

Reviewer Expertise:

Molecular biology, viral metagenomics and bioinformatics.

I confirm that I have read this submission and believe that I have an appropriate level of expertise to confirm that it is of an acceptable scientific standard, however I have significant reservations, as outlined above.

# Peer review: Reviewer response for version 1

**DOI:** 10.21956/wellcomeopenres.18969.r51813

**Published:** 2022-08-10

**Authors:** Edward Cunningham-Oakes

**Affiliations:** 1 University of Liverpool, Liverpool, UK

The study presented by Claro *et al*.

Introduction

1. Minor point, but not essential: I would encourage a quick review of punctuation/grammar for some of the longer sentences in the introduction. Alternatively, I would break up some of these longer sentences to help the reader.
2. The abbreviation CHIKV is not introduced elsewhere as Chikungunya virus before abbreviation.
3. As per previous point, LASV is not introduced as Lassa mammarenavirus before use of the abbreviation.
4. Missing word (highlighted in bold): 'SMART-9N recovered a high proportion of viral reads from **a **ZIKV isolate.'
5.  In the sentence "enabling enhanced pathogen detection for both diagnostic and surveillance application of RNA viruses", amend "diagnostic" to "diagnosis", and remove the word "application".
6. Depending on how this is published, it might be worth introducing "SMART" as (Switching Mechanism at the 5′ end of RNA Template), as you have done in the abstract.

Methods

*Sample collection*

1. "The supernatant was removed, and MEM supplemented with 2% fetal bovine serum, 1% penicillin, and streptomycin were added" - it is not necessary to add "were added" to the end of this sentence, as you have already stated "supplemented".
2. What percentage of streptomycin was the fetal bovine serum supplemented with?
3. I assume pencilin and streptomycin were added to prevent bacterial growth, but it may be worth stating why your MEM was supplemented with them.
4. Please describe the term "FFU" in full, before providing the acronym.
5. Stylistic suggestion "This sample was used to assess the performance of all three methods: multiplex PCR, SMART-9N, and Rapid SMART-9N, and the metagenomics approaches, SMART-9N, and Rapid SMART-9N, was tested in different serial ten-fold MEM dilutions up to". I would split this sentence into two, after the first mention of "Rapid SMART-9N", correct "metagenomics approaches" to "metagenomic approaches", and correct "was tested" to "were tested".
6. "with a cut-off value of Ct-values ≤ 37" -> "with a ct-value cut-off of"
7. "samples with a range of Ct-values" - can Ct-value ranges be stated in text? I know that a supplementary table is provided, but having a range in text would help readers with following the narrative without switching between supplementaries and the main text.

*Multiplex PCR*

1. Under the section "Multiplex tiling PCR", can the authors clarify what is meant by "specific bands"? Is there an amplicon size you are expecting on the gel for PCR products?

*Bioinformatics workflow*

1. The workflow presented is a robust and methodologically sound approach. However, as raised by the authors, the advantage of metagenomic sequencing is the genomic characterization of known and novel viruses in an untargeted manner, whilst this workflow uses mapping to a known reference genome, based on knowledge of what is in the clinical samples (e.g. SARS-CoV-2 nasopharangeal swabs). It would probably be of wider interest (in the discussion?) as how you would see SMART sequencing being used if you were presented with a sample with no known clinical diagnostic. I assume this would be an extension of your method to detect other RNA viruses in these samples?
2. I also noted that you used results from Kraken2 to identify a co-infection in a YFV sample, and then you obtain a reference genome to assess coverage. This is not mentioned in your methods, though it is described in the results (I also assume this would be your approach for samples with no known pathogen based on clinical diagnostics?)
3. Minor typo "samtools stats and samtools depth were used to calculate longest reads and genome coverage at 20x respectively." - a capital is needed at the start of this sentence.

Results

1. "This approach allows for amplification of RNA in the picogram input range (data not shown)" - are there plans to include this data as a supplementary? It would be exciting to see, and also extremely relevant to clinical samples, which can be of varying concentration and quality.
2. Figure 1 - no amendments to make, but I wanted to comment that this was a very helpful visual.
3. *Multiplex PCR sequencing of ZIKV isolate and YFV and SARS-CoV-2 clinical samples*
4. Figure 2 - the figure legend (and manuscript text) refers to FFU/ml, but the x-axis in panel B shows PFU/ml
5. I note that detection limits are only assessed for SMART-9N and Rapid SMART-9N methods. Do we already known the limits of detection for multiplex PCR?
6. "The sequenced YFV samples (n=21) had a median Ct-values = 25.57, between 5 and 37 generated in one 24 barcoded library." - can the authors check this sentence, as I could not quite follow it?
7. Minor/optional point: I would keep the format ranges for genome copies consistent. In the section describing YFV, you go from low to high, (1.00e00 to 1.50e10), but here, you go from high to low (2.40e05 to 1.30e02). I would go from low to high.
8. Minor typo: with an average depth **of **between 821.77x to 1570x

 

*SMART-9N and Rapid SMART-9N of ZIKV isolated-culture samples and limit of detection*

1. "and submitted to SMART-9N" - subjected to?
2. "The coverage depth was up to 10010x, and 154.25x with 6e00 FFU/mL of material, compatible with single-cell assays." - do you mean ranged between? Up to implies one of these numbers is the upper limit, but you wouldn't usually report the other if you are describing the upper limit in this way.
3. "The median N50 was 1.7kb and when the readings" - should this be "reads?"
4. Should "ZIKV genome covers" be "ZIKV genome coverage?"
5. "and the N50 2.27kb" -> "and the N50 **was** 2.27kb"
6. "with 6e07 of material input" - please state units. Was this FFU/mL?
7. *SMART-9N and Rapid SMART-9N of YFV clinical samples*
8. *"*The average coverage depth revealed higher genome depth and better coverage pattern across the genome for the metagenomics methods when compared to the multiplex PCR method." - presumably, the multiplex PCR method was specific to YFV. Do the authors know have any thoughts as to why performance is worse with a pathogen-specific assay?
9. Minor typos/sentence structure issue: "Comparable genome coverage 20-fold across the Ct-values between all methods is presented in Figure 3B" - could the authors look at this sentence again, as it is a little bit hard to interpret.

*Rapid SMART-9N of SARS-CoV-2 clinical samples*

1. Would the authors be able to clarify as to why SARS-CoV-2 were only subjected to Rapid SMART-9N?
2. "The median revealed was 91.59%, minimum 84.49%, and the Rapid SMART-9N 99.79%, minimum of 99.57%." I assume the first two values are for multiplex PCR, and the final two for Rapid SMART-9N, but this could be made clearer.
3. *Detection of other RNA viruses in clinical samples and Kraken classification*
4. *"*This allowed for the identification of a dsDNA virus genus Pa6virus, family *Siphoviridae* present in one YFV sample" - you mention (at the end of this section), the proportion of viral reads in other samples. Was there a reason that you chose to report on the Pa6virus in a YFV sample, as opposed to other identified viral reads in other samples? Was this the only evidence of putative co-infection? It may be worth noting your rationale for choosing this sample in-text.

Discussion

 Please see my note in methods regarding bioinformatic workflow in samples with no known clinical diagnostic result.

Is the rationale for developing the new method (or application) clearly explained?

Yes

Is the description of the method technically sound?

Yes

Are the conclusions about the method and its performance adequately supported by the findings presented in the article?

Yes

If any results are presented, are all the source data underlying the results available to ensure full reproducibility?

Partly

Are sufficient details provided to allow replication of the method development and its use by others?

Yes

Reviewer Expertise:

Metagenomics, microbial genomics, bioinformatics, pathogen detection

I confirm that I have read this submission and believe that I have an appropriate level of expertise to confirm that it is of an acceptable scientific standard, however I have significant reservations, as outlined above.
